# The Effectiveness of Cognitive-Behavioral Therapy in Helping People on Sick Leave to Return to Work: A Systematic Review and Meta-analysis

**DOI:** 10.1007/s10926-023-10116-4

**Published:** 2023-04-17

**Authors:** Huaying Xu, Jinxuan Cai, Rakshat Sawhney, Stephen Jiang, Nicholas Buys, Jing Sun

**Affiliations:** 1 School of Medicine and Dentistry, Griffith University, Nathan, Q4215 Australia; 2 Centre for Work, Organisation and Wellbing, Griffith University, Nathan, Q4215 Australia; 3 Institute for Integrated Intelligence and Systems, Griffith University, Nathan, Q4215 Australia

**Keywords:** Sick leave, Return-to-work, Cognitive behavioural therapy, Musculoskeletal disease, Depression

## Abstract

**Purpose:**

Previous research has systematically studied the effectiveness of Cognitive Behavioral Therapy (CBT)-based interventions in managing both mental and physical symptoms of chronic disease including depression, stress-related mental disorders (SMD), and chronic pain that are common causes of sick leave. However, a systematic review focusing on the effectiveness of CBT in facilitating RTW is lacking. This study compiles research on utilizing CBT-based interventions for helping employees on sick leave return to work.

**Methods:**

Randomized controlled trials (RCT) published between 1 January 1990 and 27 June 2022 were searched in MEDLINE, EMBASE, The Cochrane Library, Scopus, PsycINFO, Web of Science, and PubMed. The primary outcome variables included a return to work (RTW) measure and sickness absences. The secondary outcomes include psychological conditions (mental illness, stress, anxiety, and depression) and physical condition (working ability, fatigue, and physical function).

**Results:**

Thirty-four RCTs were included in the analysis. Fifteen RCTs with 1727 participants reported on sick leave. Results showed that participants who completed CBT intervention had reduced sick leave in days (mean reduction − 3.654; 95%CI − 5.253, − 2.046; p < 0.001) compared to the control group. Sixteen papers with 2298 participants reported that the intervention group RTW 1.5 days earlier (95%CI 1.019, 1.722; p < 0.05). CBT-based interventions were effective in managing fatigue, mental illness, and depression, and improving physical function while it showed no effects in managing stress, anxiety and working ability.

**Conclusions:**

The findings indicate that CBT-based interventions are effective in reducing the length of sick leave and facilitating the RTW of employees in the intervention group.

## Introduction

The Sickness Absence Dictionary defines sick leave as contract employees or self-employees taking days off from their regular work to deal with their personal illness [1]; it is granted based on the different policies in each country. Long-term sick leave is defined as taking a long period of consecutive days off, and the number of days used for long-term sick leave is directly associated with the social insurance programmes in different nations [1]. In most countries including Sweden, Australia and Finland, long-term sick leave is defined as taking more than 60 consecutive days or 3 months off from work in 12 months [1].

Numerous reasons exist for sick leave beyond physical or mental medical conditions [1, 2]. Sick leave can be caused by factors including poor working environment and poor relationships between colleagues and supervisors at work, resulting in significantly economic loss. Among the 15 member states of the European Union in 2000, an average of 14.5% of people had taken at least 1 day of sick leave [3]. In Denmark, 3.6% of all work hours are lost due to sick leave [4]. Sick leave has resulted in a loss of 175 million working days in Britain in the year 2006 which is the equivalent of seven days for each working person [5], and it significantly contributes to low productivity, with every 1% increase in the rate of sickness leave resulting in a productivity loss of 0.66% [6].

Mental health disorders including depression and SMD are the leading causes of sick leave. In Sweden, mental health disorders accounted for 53% of sick leave causes in women and 42% of sick leave causes in men in the year 2019 [7]. The clinical features of depression (e.g. insomnia, anhedonia, feeling of worthlessness, fatigue, diminished concentration), and the poor psychological and behavioural characteristics present among SMD patients can contribute to poor working ability leading to sick leave [8]. The economic cost of mental health disorders in Europe is estimated to be $610 billion per year, with the majority arising from reduced employment and lost productivity [9]. Chronic musculoskeletal (MSK) pain is the second most common cause of sick leave [10]. Chronic MSK pain is defined in this study as pain that persists for more than three months and may occur anywhere in the body. It can be related to injuries to muscles, bones, ligaments, tendons, and/or nerves [11]. As an example, the productivity loss due to chronic lower back pain alone in the United States was up to 28 billion USD [12]. Chronic MSK pain is often related to muscle overuse, repetitive muscle inflammation from muscle strain, and heavy weight lifting [13]. Chronic MSK pain is relevant to all types of work, such as office work and building construction.

RTW interventions have been trialled to help employees on sick leave restore their physical and mental capacities and to avoid the recurrence of symptoms. Studies show that the symptomatic treatment of common mental disorders and MSK pain alone may be insufficient to reduce sick absence partly due to unadjusted working habits after RTW [14]. Recent studies have trialled various forms of cognitive behavioural therapy (CBT) to help employees return to work and adjust to working habits after RTW [15, 16]. There is strong evidence supporting the benefits of CBT as a treatment for a wide range of psychological conditions, such as anxiety and depression, which may help employees with sick leave to return to work early [17–21]. CBT works by training people to be aware of their thoughts and behaviours and to replace negative instinctive reactions to various daily life scenarios with more positive outlooks thereby allowing them to modulate their symptoms [22, 23]. The behavioural component employs various techniques, including reinforcement, classes and shaping, to improve employees’ functional status and help them develop positive relationships with work [24, 25]. The cognitive component helps individuals to identify and link their obstructive thoughts, problematic behaviours, and negative feelings together [23].

CBT is problem-oriented and focuses on improving the patient’s current cognition and thought processes [25]. Therefore, CBT is a useful intervention model for the management of chronic problems where there is no immediate cure [25]. Thus, CBT is a strong candidate when considering treatment options for people taking long-term sick leave because it can help manage the behaviours and thought processes underlying their reasons for taking the sick leave in the first place. Although CBT is primarily focused on the treatment of mental illnesses, applying the principles of CBT with a focus on positive reinforcement and behavioural changes may help improve resilience and the ability to cope with the pain, thereby increasing work satisfaction and reducing the mental burden of MSK-based reasons for long-term sick leave [26]. CBT is already the first-line treatment for a variety of psychological illnesses and can be utilised to great effect in the management of stress and burnout in the workplace [17, 27]. Thoughts and behaviours in the workplace contribute greatly to both the development and alleviation of stress, which can then lead to burnout [28]. CBT could be utilised to great effect in the control of these factors to help employees return to work and increase productivity.

The effectiveness of CBT in reducing the duration of sick leave has been investigated in various RCTs across different countries, albeit with inconsistent results. Previous RCTs have shown that CBT-based treatment can significantly reduce the length of MSK-related sick leaves and is effective in improving the physical and mental health of employees [20, 21, 29]. Other similar interventions have also been shown to have a significant effect on helping employees return to work after taking sick leaves due to mental conditions including stress and burnout [30, 31]. However, results from various studies are inconsistent with some RCTs showing that although CBT-based treatment led to improved outcomes compared to no treatment, there was no significant difference between the proposed treatment and care as usual [32–35]. In summary, low sample size, homogeneity of the sample population, and the differences in control groups may account for these inconsistencies among different studies. Although there are systematic reviews and meta-analyses investigating interventions supporting RTW among workers with different reasons for sick leave, a systematic review focusing on the effectiveness of CBT in facilitating RTW is lacking [36–39]. To fill in this research gap, this study aimed to conduct a systematic review and meta-analysis of RCTs to evaluate the effectiveness of CBT in helping employees with sick leave RTW and in reducing stress, anxiety, depression, fatigue, mental illness, and improving working ability and physical health.

## Methodology

### Literature Search

The study was registered at PROSPERO (Registration Number: CRD42021260666). The search was conducted in the following electronic databases: MEDLINE, EMBASE, The Cochrane Library (Cochrane Database of Systematic Reviews, Cochrane Central Register of Controlled Trials (CENTRAL), Cochrane Methodology Register), Scopus, PsycINFO, Web of Science, PubMed, and Chinese Zhi Wang. Then, the keywords were put in the Thesis and Dissertation to include the unpublished articles. Searches were confined to literature published from January 1, 1990 to June 27th 2022 by using the following key terms: (“ Employee OR employees OR worker OR workers OR staff OR personnel’ [All fields]) AND (“ Sick OR sick leave OR ill OR illness” [All fields]) AND (“ Return to work OR go back to work OR resume work OR part time OR full time OR casual OR temporary work” [All fields]) AND (“ Cognitive behaviour therapy OR CBT OR cognitive behaviour therapy” [All fields]) AND (“ Randomised controlled trials Or Randomised controlled trial OR Randomized controlled trials OR Randomized controlled trial OR RCT” [All fields].

### Inclusion Criteria

The inclusion criteria for this systematic review and meta-analysis was developed based on the PICO approach [40]:

*Participants*: Participants were employees at a workplace and on sick leave or leave due to a workplace incident. All participants must be 18 years or above.

*Intervention*: The study design was RCT or RCT equivalent studies. The intervention must be based on CBT with both cognitive and behavioural therapy as compulsory components in combination with common CBT techniques including homework assignment, stress management, relapsing preventions, problem-solving strategies, and rehabilitation. The intervention can be a single CBT or CBT-based multiple programme intervention in combination with other managements including motivational interviews, and graded activity. CBT can be delivered individually or in groups in the form of face-to-face or online (online modules are included).

*Control*: Control group can be anything including non-CBT interventions such as care-as-usual, no treatment, graded activity, Qigong that refers to a traditional Chinese mind-body exercise to improve health by relieving stress, anxiety and improving physical health, work rehabilitation, and conventional care.

*Outcome*: The study outcome includes whether the employee return to work or not, and the number of days for sick leave. The secondary outcomes included stress, fatigue, mental illness, anxiety, depression, working ability and physical function.

The exclusion criteria for this review were:

*Participants*: People who were below the age of 18 OR participants and not employees or the employees were not on sick leave

*Intervention*: The intervention is not based on CBT.

*Control*: Papers will be excluded if there is no control group.

*Outcome*: The outcome is not whether the employee returned to work or sick leave.

### Data Extraction

The titles and abstracts of non-duplicated papers that were selected during the search were independently screened by two authors to identify papers that potentially met the inclusion criteria. If there was any disagreement about the inclusion or exclusion of a paper between two authors, the paper was evaluated by a third author for a conclusive decision. Full texts of eligible studies that met the requirement of qualitative assessment were assessed for data extraction. Data extraction for study characteristics included country, publication year, study design (single-blinded, double-blinded, no blinded RCT), study population (proportion intervention and control employee participants), participant demographics (including age, the ratio of gender per group, number of employees with RTW in the post-intervention phase), type of interventions (characterization of the intervention group in addition to the control group), control group activity, duration of intervention, primary outcomes that were the number of people RTW and sick leave, secondary outcomes including psychological condition (mental illness, stress, anxiety, and depression) and physical condition (working ability, fatigue, and physical function), and quality assessment score. Missing data were requested from the corresponding author of the study. If sickness absences were measured in weeks, the number of days for sickness absences was calculated by multiplying the number of weeks by 5. The subgroup analysis included the delivery method of CBT (face to face vs. remote), an education level (< 9 years vs. 9–12 years vs. > 12 years), reasons for sick leave (MSK vs. psychological vs. others), combined with mood symptoms (no vs. yes), type of intervention (single CBT vs. combined CBT), duration of sessions (< 90 min vs. ≥ 90 min), treatment duration (< 16 weeks vs. ≥ 16 weeks), treatment form (individual session vs. group session vs. mixed), study design (non-RCT vs. RCT), and the components of CBT including (i) rehabilitation service utilization (no vs. yes), (ii) support from supervisor (no vs. yes), (iii) mood management (no vs. yes), (iv) stress management (no vs. yes), (v) homework assignment (no vs. yes), (vi) psychological education (no vs. yes), (vii) relapse prevention (no vs. yes), (viii) interpersonal strategies (no vs. yes).

### Quality Assessment

Papers that met inclusion criteria were independently screened for risk by two authors. PEDro method was used for evaluating the risk assessment that was evidenced as a valid measure of the methodological quality of clinical trials [40]. PEDro scale consists of 10 ‘yes’ or ‘no’ questions. Every ‘yes’ to the question is worth 1 point. It categorizes the quality of papers into three-level: high quality with 8 points or above, moderate quality with 4–7 points, and low quality that has less than three points [41]. The criteria for the application of PEDro scale include random allocation of subjects, concealed randomization, the similarity of baseline information between groups, blinding to subjects, assessors, and researchers, attrition rate, maintenance of group allocations, use of intention-to-treat analysis, use of variability measures, and use of between-group comparison methods [40]. Low-quality papers (3 points) were automatically excluded.

### Statistical Analysis

Outcome data extraction was conducted and extracted data contained information on data variable type, mean scores with SDs, and confidence interval. The primary outcome included an RTW measure (yes/no, categorical variable) and sickness absences (continuous variable). The secondary outcomes include psychological conditions (mental illness, stress, anxiety, and depression) and physical condition (working ability, fatigue, and physical function). RTW variable was presented in numbers and sample size per group. Continuous variables such as sickness absences, listed psychological and physical conditions were presented as mean and standard deviation.

The effect size for all outcome variables was measured by the random-effects model. A p-value of < 0.05 was considered statistically significant. Publication bias was assessed by Egger regression analysis. A P-value of less than 0.05 would suggest a publication bias, and further sensitivity analysis will be conducted to validate it.

I^2^ describing the variations across the study was calculated for the assessment of heterogeneity. An I^2^ > 50% meant a significant effect of CBT on the outcome variables. In such conditions, further subgroup analyses were conducted for outcome variables including sick leave and depression. The subgroup analysis was conducted in relation to variables including delivery method of CBT (face to face vs. remote), an education level (< 9 years vs. 9–12 years vs. > 12 years), reasons for sick leave (MSK vs. psychological vs. others), combined with mood symptoms (no vs. yes), type of intervention (single CBT vs. combined CBT), duration of sessions (< 90 min vs. ≥ 90 min), treatment duration (< 16 weeks vs. ≥ 16 weeks), treatment form (individual session vs. group session vs. mixed), study design (non-RCT vs. RCT), and the components of CBT including (i) rehabilitation service utilization (no vs. yes), (ii) support from supervisor (no vs. yes), (iii) mood management (no vs. yes), (iv) stress management (no vs. yes), (v) homework assignment (no vs. yes), (vi) psychological education (no vs. yes), (vii) relapse prevention (no vs. yes), (viii) interpersonal strategies (no vs. yes).

## Results

### Search Results

**Fig. 1:**
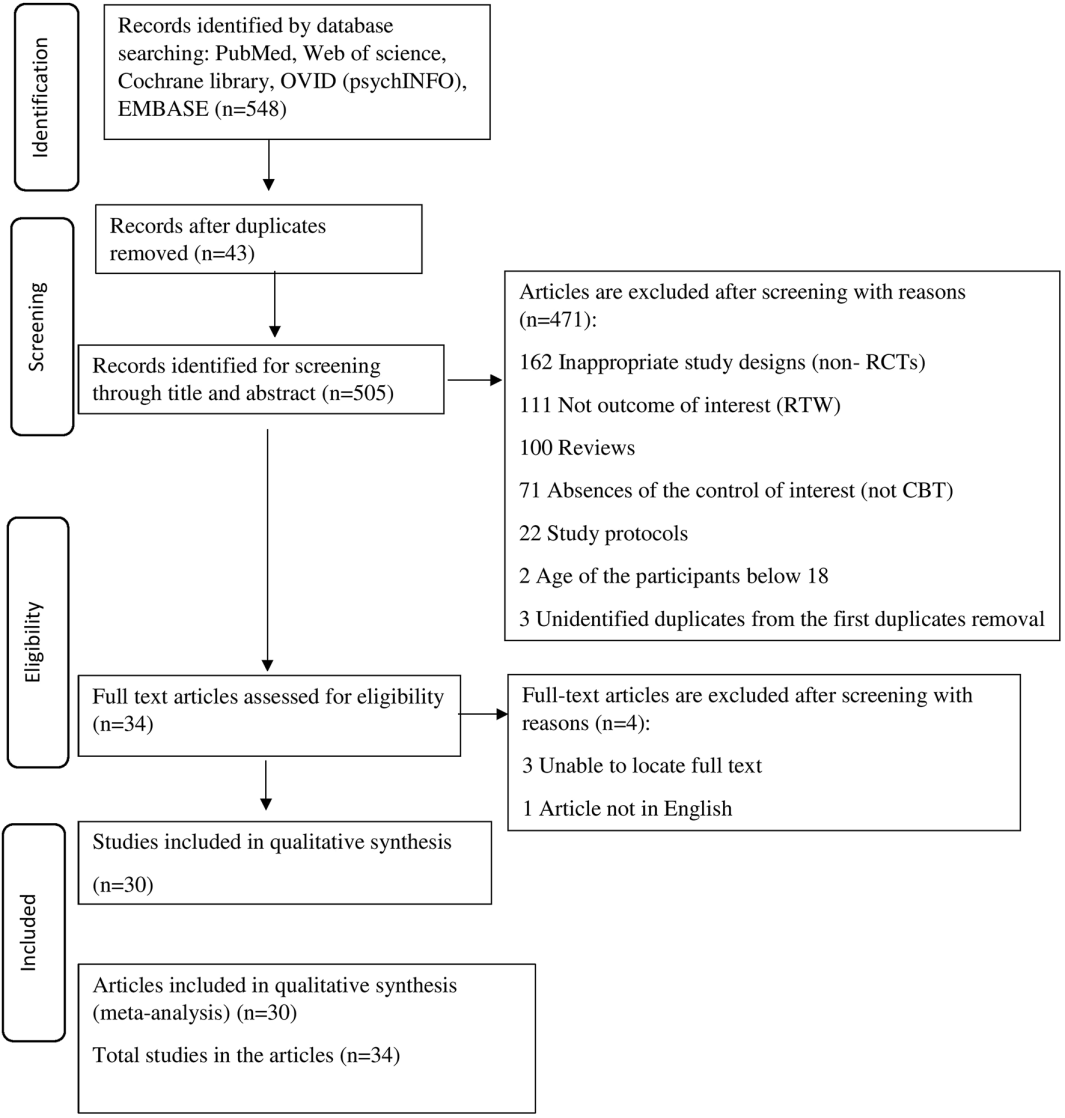
PRISMA flow diagram

As seen in Fig. 1, 548 articles were identified by two assessors through the initial search and 43 articles were removed due to duplication. 471 articles were further excluded by screening abstracts and titles. The full text was retrieved for the remaining 34 articles, and they were assessed according to the inclusion and exclusion criteria. In the end, 30 RCTs or RCT equivalent studies were included in this systematic review and meta-analysis.

### Characteristics of Included Studies

Individual analyses were performed for three studies that investigated the effectiveness of both individual CBTs and combined CBTs, and one study that investigated the effect of CBT on both individuals and work groups. Combined CBT refers to multimodal interventions that utilised CBT with a work-focused purpose in combination with other strategies including motivational interviews, functional capacity training modules and workplace-based interventions including occupational adjustment. Therefore, there were 34 effective comparing groups with a total of 6065 participants included. These studies were primarily done in developed countries including USA [42], Germany [43–46], Netherland [33, 34, 47–51], Denmark [30, 52, 53], Sweden [16, 19, 21, 31, 32, 54–59], Norway [35, 60], Canada [61, 62], Spain [20]. Out of the 34 studies, 21 studies were designed for patients on sick leave due to psychological reasons [16, 30–34, 42, 44, 45, 48, 50–52, 54, 55, 58–60], 12 studies for patients on sick leave due to MSK issues [19–21, 35, 43, 46, 47, 49, 53, 56, 57, 61, 62] and 1 study for patients due to other reasons such as unexplained fatigue [47]. 16 trials used CBT combine with other treatments/therapies [16, 30, 31, 35, 42, 43, 46, 49–51, 54, 55, 58–62] including motivational interviews, problem solving skills, education, and care as usual, while 18 trials used only CBT as an intervention [16, 19, 20, 32–34, 44, 45, 47, 48, 52, 53, 56–58] (Table1). 20 trials conducted individual based-CBT interventions [16, 20, 32–34, 42, 44, 45, 47, 50–52, 54, 58–62], 11 trials conducted group based-CBT interventions [19, 21, 30, 31, 33, 43, 48, 53, 55–57], and 3 trials delivered CBT through a mixed mode [35, 46, 49] with both individual and group sessions. Most CBTs were delivered face to face while 5 trials were delivered remotely [20, 32, 42, 51, 54] through strategies including online learning modules (Table1). 21 trials used usual care as control [16, 19–21, 32, 33, 35, 42, 45, 47, 48, 50, 51, 54–56, 58–62], 8 trials used no treatment [19, 30, 34, 44, 52, 53, 56, 57] and 5 used non-CBT intervention (conventional care [43, 46], Qigong [31], work rehabilitation [46], and graded activity [49]) (Table1).

**Table 1:** Full details of studies used in meta-analysis

Study	Participants (MSK/Psychological/Other)	Location of the study	Study design (single blind = NB/SB/DB/TB)	Mean age	Gender ratio for intervention (M/F)	Gender ratio for Control (M/F)	Duration of intervention	CBT intervention (what does the intervention group do, in addition to control group)	Control group activity (what they do)	Outcome variables	Quality of assessment
Adler et al. [42]	Psychological	USA	NB	54.6	24|61	20|62	16 weeks	8 × 50 min sessions twice a month; The Work-focused intervention has three integrated modalities including work-focused CBT. Each one addresses a specific barrier to effective functioning and stresses the acquisition of self-care strategies through a combination of “homework” assignments, counsellor feedback and motivational interviewing to optimize functional outcomes using vocational, medical, and psychological strategies	Usual care for dysthymia	RTW, depression	7
Bethge et al. [43]	MSK	German	NB	48.9	26|92	34|84	3-week	3-week Multimodal work hardening group programme (CBT-based) including 6 modules: 1 work and health; 2 occupational competences; 3 exercise; 4 aquatic exercise; 5 functional capacity training; 6 relaxation	Conventional musculoskeletal rehabilitation without CBT-based learning modules at the same rehabilitation centre; Treatment primarily included exercise therapies, patient education (e.g. pain management and nutrition counselling) and psychosocial interventions (e.g. psychological and social counselling)	Work status; depression; anxiety; pain cognition, mental health	6
Blonk et al., 2006 [36] (combined intervention)	Psychological	Netherlands	NB	42	32	33	11 weeks	The combined intervention (CI) consisted of five to six sessions of approximately an hour, twice a week. These individual sessions were held at home or at the workplace of the self-employed and were conducted by a labour expert. Six labour experts participated in the study and were trained in brief CBT-based stress management. The stress-management part of the intervention consisted of psychoeducation on work stress, registration of symptoms and situations, relaxation, self-help books on rational emotive behaviour therapy, and time-management and writing assignments. At the end of each session, homework assignments were given concerning these topics. For example, participants were asked to read a self-help booklet on rational emotive behaviour therapy and to perform specific exercises described in the booklet. In the following session, these exercises were discussed in combination with new topics and exercises. In addition to the stress-management part, the labour experts gave advice on work processes and provided suggestions on how to lower the workload and job demands and increase the decision latitude, if necessary	No-treatment control group; The first session with the GP was shortly after the initial sick leave The aim of this session was primarily to check the legitimacy of the work-disability benefit The second session was held after approximately 4 months and had the same aim as the first session	Depression; Anxiety; Stress; Sickness absence	6
Blonk et al., 2006 [34] (Individualised CBT)	Psychological	Netherlands	NB	42	33	33	11 weeks	CBT consisted of 11 two-weekly individual sessions of approximately 45 min per session. Psychologists followed a highly structured protocol. The first six sessions focused on cognitive restructuring and on registration of symptoms and situations. The following five sessions focused predominantly on a further expansion of cognitive restructuring. For such sessions, one of the following six modules is usually used: cognitive restructuring, work resumption, time-management, workplace interventions, conflict handling, and fatigue. All sessions started with a discussion of the registration assignment and ended with either a continuation of previous assignments or an extension of the assignments with new ones. In all modules, with the exception of the cognitive restructuring and fatigue modules, the assignments were related to the work situation	No-treatment control group; The first session with the GP was shortly after the initial sick leave The aim of this session was primarily to check the legitimacy of the work-disability benefit The second session was held after approximately 4 months and had the same aim as the first session	Depression; Anxiety; Stress; Sickness absence	6
Dalgaard et al. [52]	psychological	Denmark	SB	45	15|43	12|37	16 weeks	The intervention consisted of six, one-hour sessions with individual work-focused CBT conducted by a psychology gist over 16 weeks. This involved: (i) identifying work-related stressors, (ii) modifying cognitive and behavioural coping strategies, (iii) providing psychoeducation about work related stress, (iv) assigning homework, and (v) assisting participants in planning RTW	Clinical assessment but no treatment	Length to RTW	7
Eriksson et al. [55]	Psychological	Sweden	NB	36.3	17|35	10|28	12 weeks	7 online self-learning modules + 3 phone calls over 8–12 weeks	Usual care (received the treatment typically provided for depression)	RTW, sick leave, depression	6
Glasscock et al. [44]	psychological	Germany	NB	45	9|48	13|67	16 weeks	6 × 60 min individual CBT over 16 weeks + offer of participation of psychologist in meeting between patient and employer; early sessions involved psychoeducation concerning the nature of stress and patients were introduced to a stress model, which forms the theoretical basis of the intervention. Later sessions included a focus on what the patient could do, once treatment was over, to prevent relapse	No treatment; Followed up only with questionnaires	Stress level; GHQ-30; RTW	7
Haldorsen et al. [35]	MSK	Norway	NB	43	112|200	59|98	4 weeks	6 h session × 5 days per week × 4 weeks; the programme included physical treatment, CBT, education, and workplace-based interventions. The treatment was given as partly individual (based on diagnosis and the pre-clinical result) and partly group activities (morning exercise, body awareness training, relaxation training)	Receive normal physiotherapy from GP consultations	Pain; General health; Distress, RTW	7
Heiden et al. [56]	Psychological	Sweden	NB	44	28	23	10 weeks	2 × 3-h group sessions per week for 10 weeks; The sessions contained educational elements in the form of seminars, group discussions, and required daily practice of skills	Participants in the physical activity group were offered 2 exercise sessions per week for 10 weeks. One of the sessions followed a rehabilitation programme with low-intensity exercises in a warm water pool	RTW	6
Huibers et al. [47]	Other	Netherlands	NB	43.5	37|39	31|44	16 weeks	5–7 × 30 min over 4 months; the intervention consisted of 2 stages: (1) assessment of perpetuating factors such assessment of perpetuating factors such as cognition, positive behaviour, social factors; (2) modification of identified perpetuating factors by setting up goals, providing helpful cognition etc	No research intervention was offered to patients in the control group. Patients in either group were free to visit their regular GP for usual care	Physical function; distress; RTW; Sickness absence	6
Jensen et al. [19]	MSK	Sweden	SB	43.8	27|22	20|28	4 weeks (The study ran for 4 weeks, and the data was collected at 36-month follow-up.)	13-14 h group session/week for 4 weeks; The CBT program included activity planning and goal setting, problem solving, applied relaxation, cognitive coping strategies, activity pacing, the role of vicious circles and how to break them, the role of Significant others and assertion training, and individually tailored homework assignments	Treatment as usual (= no treatment, normal routines in health care)	Short Form 36 for general health; Perceived relevance of rehabilitation; work absence; body pain; mental health	6
Jensen et al. [57]	MSK	Sweden	SB	43	27|22	20|28	4 weeks (The study ran for 4 weeks, and the data was collected at 36-month follow-up.)	13-14 h group session/week for 4 weeks; The CBT program included activity planning and goal setting, problem solving, applied relaxation, cognitive coping strategies, activity pacing, the role of vicious circles and how to break them, the role of significant others and assertion training, and individually tailored homework assignments	Treatment as usual (= no treatment, normal routines in health care)	Absence from work; Short Form-36 (SF-36) for mental health	6
Jørgensen et al. [53]	MSK	Denmark	NB	45.5	0|99	0|100	52 weeks	Group discussion with 2 phases (single CBT); phase I: The first intensive intervention phase consisted of a 2-h session at the workplace twice a month. The cognitive behavioural training mainly comprised group discussions of issues regarding pain-related dysfunctional attitudes, coping and management, with facilitation of functional alternatives. In the second phase, the number of training sessions was gradually reduced, with only one session of 1 h’s duration per month during the last 6 months. In this phase, the experiences, and considerations of the cognitive and behavioural changes of the participants from the first phase were debated, and reflections on and support for obtaining long-lasting cognitive and behavioural changes were the focus. (2 h/session for twice a month; one hr/session, once per month)	1 h health check without any intervention (reference group)	Work ability; Sickness absence; Pain	6
Kaldo et al. [72]	Psychological	Sweden	NB	41.9	62|171	61|169	12 weeks with 52 weeks (1 year) follow up	The treatment was based on 34 self-help text modules, each based on established CBT principles and presenting information on a specific problem area, useful methods to handle it and an online homework report. Patients worked with each module for about a week with brief but active support from a therapist: a clinical psychologist or last-year psychology student under supervision	GP standard care for depression	Sickness absence; work ability	7
Kroger et al. [45]	Psychological	Germany	NB	41.85	8|5	6|7	24 weeks	W-CBT:(a) In providing the individual treatment model, the workplace was regarded as a resource of self-efficacy and self-worth. (b) When problems arose in the workplace, they were identified and tackled within the framework of problem-solving training. (c) The skills successfully applied in the workplace were transferred to other problematic areas (e.g., also granting oneself a time-off at home). (d) For all patients, a plan for reintegration was developed on a form and its implementation was therapeutically supported. (e) In this respect, hurdles that arose during the reintegration phase were identified and removed where possible (e.g., disputing dysfunctional thoughts and changing physical working conditions). (f) The occupational health physicians and the employees’ superiors were included in the therapy if possible	CBT-AU: therapists were not allowed to conduct any work-related assessments or interventions; followed a standard German manual for depression treatment	Sickness-leave absence; depression (BDI)	7
Lambeek et al. [62]	MSK	Canada	NB	46.2	37|29	41/27	64 weeks	Integrated care included: usual care + care by occupational physician + workplace intervention + up to 26 sessions of graded activity (CBT based)	Usual care from GP for back pain	Time to sustainable RTW, pain intensity, physical function	7
Lagerveld et al. [48]	Psychological	Netherlands	NB	40.75(10)	41|48	26|53	24 weeks	W-CBT (12 sessions over 6 months) consisted of the regular treatment (CBT) plus a module focusing on work and the return to work; W-CBT treatment consisted first of specific work-related (homework) exercises/interventions that were additional to regular CBT interventions (such as drawing a RTW plan). Second, regular CBT interventions or exercises were framed as much as possible in the work context (such as work-focused psychoeducation or work-focused behavioural experiments to challenge dysfunctional thoughts). In addition to these two work-related components, treatment time could also be spent on nonwork issues (e.g., marital problems)	R-CBT: treatment for work-related common mental disorders in Netherland	Full RTW (%); No. of days to fully RTW/partially RTW; depression, anxiety, stress, burn out	5
Leon et al. [20]	MSK	Spain	NB	44.7	22|93	19|47	96 weeks	The individual CBT was provided weekly in 3 levels, according to the patients’ evolution. All patients received the first level, consisting of 2 60-min Early Cognitive–Behavioural Treatment for MSD. The first session included education on pain and ergonomics and training in abdominal breathing. In the second session, doubts, and difficulties with the booklet in the first session were resolved. After 2 weeks, patients who continued sick leave went to the second level, which was composed of 3 sessions. Patients who did not return to work after that started in the third level of the cognitive–behavioural treatment, which had an indeterminate number of sessions depending on the patient’s evolution. In this level, a revision of the previous techniques was performed, and patients were trained in coping skills for interpersonal and work issues. Follow-up lasted 6–24 months	Rheumatologic care program	Sickness absence	7
Linton et al. [58]	MSK	Sweden	NB	48	13|56	3|44	6 weeks	The intervention encompasses 6 group sessions where participants meet in groups of 6 to 10 people, 6 times, once a week for 2 h. Each session has several parts: (1) 15 min for setting the tone for the session as well as to review homework; (2) 15 min to introduce the topic of the session and to provide relevant facts; (3) 30 min for problem solving in pairs; (4) 30 min fro skills training; (5) 15 min for homework assignment and discussion	Minimal treatment group: provided with physical examination and self-care booklet	Sickness absence; Stress; Depression; Anxiety; Pain; Physical function	4
Marhold et al. [26]	MSK	Sweden	NB	46	0|18	0|18	12 weeks	CBT focusing on pain coping skills and application (12 weekly group sessions × 2.5 h + 2 booster sessions in 1 and 3 months after the intervention); During the first six weeks of the program, participants were educated on the gate control theory of pain and the model of risk factors and coping in cooperation of the strategies including goal settings, graded activity training, pacing of activities and cognitive techniques and stress management. During the rest six sessions, different pain coping skills were taught to the patients as in traditional cognitive ± behavioural pain management. The last six sessions concentrated on helping the patients to return-to work and teaching them how to apply the pain coping skills to various occupational risk factors at their workplaces	Usual care including visiting physician, physiotherapist, a nurse etc	Sickness absence; Pain; Depression; Physical function	7
Reme et al. [61]	Psychological	Norway	NB	40.4	193|437	196|365	52 weeks	15 sessions of combined CBT: The individual job support was based on the ‘Individual Placement and Support (IPS)’ approach, developed for people with severe mental illness,18 and was offered to those in need of individual job support (primarily participants on long-term disability) to facilitate workplace adaptations or identification of appropriate employment. IPS represents a relatively new approach to vocational rehabilitation and incorporates the following eight principles: eligibility based on consumer choice, focus on competitive employment, integration of mental health and employment services, attention to client preferences, work incentives planning, rapid job search, systematic job development and individualised job support. The IPS framework is less specific on choice of therapeutic approach within the mental health services	Care as usual	Increased or maintained work participation (= No. of RTW), anxiety, depression and stress	7
Salomonsson et al. [60] (combined CBT)	Psychological	Sweden	NB	41.9	13|67	10|54	25 weeks	starting with three RTW-I sessions (the first three modules), followed by CBT for the specific disorder where a brief follow-up on the RTW progress was added at the end of each session. Depending on the specific disorder and CBT protocol, the COMBO treatment thus varied between 10 and 25 sessions during a period of maximum 25 weeks	Treatments were based on available evidence-based CBT protocols for each specific. The length of CBT varied between 8 and 20 weekly sessions disorder	Sick leave; sick leave status (part time sick leave; full time sick leave; no sick leave)	7
Salomonsson et al. [60] (WORK focused CBT)	Psychological	Sweden	NB	42.35	14|53	10|54	25 weeks	The treatment consisted of four central modules: (1) conceptualisation, (2) psychoeducation, (3) planning and (4) monitoring. These modules were worked through in 10 sessions over a period of 20 weeks, initially weekly then follow-ups more sparsely. In the conceptualisation phase, the causes for sick leave were examined, as well as work-related goals and perceived barriers to return-to-work. In the psychoeducation module, information was given about potential pros and cons with sick leave, the national social security system and medical guidelines for prescribing sick leave. In the planning module, therapist and patient formulated a plan for RTW, which was agreed with the employer, the patient’s general practitioner and the social insurance agency. In the final module, focus was on monitoring the steps taken and supporting the patient in dealing with difficulties	Treatments were based on available evidence-based CBT protocols for each specific disorder	Sick leave; sick leave status (part time sick leave; full time sick leave; no sick leave)	7
Salomonsson et al. [59] (combined CBT)	Psychological	Sweden	NB	42.4	6|45	6|46	25 weeks	The treatment consisted of four central modules: (1) conceptualisation, (2) psychoeducation, (3) planning and (4) monitoring. These modules were worked through in 10 sessions over a period of 20 weeks, initially weekly then follow-ups more sparsely. In the conceptualisation phase, the causes for sick leave were examined, as well as work-related goals and perceived barriers to return-to-work. In the psychoeducation module, information was given about potential pros and cons with sick leave, the national social security system and medical guidelines for prescribing sick leave. In the planning module, therapist and patient formulated a plan for RTW, which was agreed with the employer, the patient’s general practitioner and the social insurance agency. In the final module, focus was on monitoring the steps taken and supporting the patient in dealing with difficulties	Treatments were based on available evidence-based CBT protocols for each specific disorder	Stress, depression, anxiety for stress related disorders	7
Salomonsson et al. [59] (work focused CBT)	Psychological	Sweden	NB	42.7	8|41	6|46	25 weeks	The treatment consisted of four central modules: (1) conceptualisation, (2) psychoeducation, (3) planning and (4) monitoring. These modules were worked through in 10 sessions over a period of 20 weeks, initially weekly then follow-ups more sparsely. In the conceptualisation phase, the causes for sick leave were examined, as well as work-related goals and perceived barriers to return-to-work. In the psychoeducation module, information was given about potential pros and cons with sick leave, the national social security system and medical guidelines for prescribing sick leave. In the planning module, therapist and patient formulated a plan for RTW, which was agreed with the employer, the patient’s general practitioner and the social insurance agency. In the final module, focus was on monitoring the steps taken and supporting the patient in dealing with difficulties	Treatments were based on available evidence-based CBT protocols for each specific disorder	Stress, depression, anxiety for stress related disorders	7
Stenlund et al. [31]	Psychological	Sweden	NB	41.6	18|49	22|47	52 weeks	CBT: The program consisted of 30 sessions, each 3 h long and spread over 1 year, with 20 group meetings the first 6 months and ten meetings the last 6 months. The five key components of the program were (1) education (for example, stress reactions, sleep, affect, medication, the importance of rest in order to recover), (2) awareness of reactions and “selftalk”, (3) development of behavioral/cognitive/emotional skills, (4) spiritual issues and life values, and (5) preparation for return to work. + Qigong + work rehabilitation	Qigong + + work rehabilitation	Stress; Depression; Anxiety; physical function	6
Schultz et al. [63]	MSK	Canada	NB	39	56|9	19|18	24 weeks	Early intervention (CBT-based), one to one session with nurse advisor, Workplace visit by nurse advisor, communication between a worker's compensation physician and the worker's primary healthcare practitioner	Case management in the usual manner of worker's compensation system in British Columbia	Return to Work, disability risk, cost of healthcare, duration of disability	6
Schweikert et al. [46]	MSK	Germany	NB	46.7	166|34	173|36	12 weeks	6 × 1.5 h group sessions + 2 × 0.5 h individual session + usual care. The 6 group sessions, with an average group size of 6 patients (maximum 8), had the following topics: (1) onset and development of chronic LBP: physical and psychological factors in back pain; (2) role of attention in pain and means of focusing on distracting thoughts and actions; (3) stress and pain: methods of stress reduction such as cognitive reappraisal of stress stimuli; (4) social stress and pain: methods of gaining self-confidence; (5) mood and pain: ways of adopting a more positive attitude; and (6) thoughts and pain: fighting negative thoughts and attitudes such as catastrophizing	Conventional 3-week inpatient rehabilitation program in groups, consisting of daily physiotherapy in small groups, massage of spinal region, electrotherapeutical measures, 1-h seminar regarding back training, twice-daily exercise program, seminars on lifestyle, and risk factors for back pain and its process of becoming chronic	Number of days off work, quality of life, cost	6
Van den Hout et al. [49]	MSK	Netherlands	NB	40.5	33|12	31|8	8 weeks	Combined CBT including 15 × 1 h sessions for graded activity + 3 additional sessions dedicated to back education and lifting instructions, 30 min per week with occupational therapist individually, 10 × 90 min problem solving sessions (CBT based)	15 × 1 h sessions graded activity + 30 min per week with occupational therapist, 10 × 90 min education sessions	Return to Work, days of sick leave	5
Van der Klink et al. [50]	Psychological	Netherlands	SB	47.6	72|37	49|10	12 weeks	4–5 individual sessions × 90 min with occupational physician; in first 6 weeks + 1 session after work resumption; based on the three-stage model: (1) understanding the origin and cause of the loss of control. Patients were also stimulated to do more nondemanding daily activities. (2) Patients were asked to draw up an inventory of stressors and to develop problem solving strategies for these causes of stress. (3) Patients put these problem-solving strategies into practice and extend their activities to include more demanding ones. The patients’ own responsibility and active role in the recovery process was emphasised	Usual care depending on healthcare team; based on empathic counselling, instruction about stress, lifestyle advice, and discussion of work problems with the patient and company management	Return to work, duration of sick leave, 4 dimensional symptom questionnaire	7
Vente et al. [33] (individualised CBT)	Psychological	Netherlands	NB	41.2	17|11	17|9	16 weeks	12 × 1-h individual CBT-based SMT Treatment protocols for both the individual and group SMT comprised five modules (a) psychoeducation, self-assessment of stressors and complaints, lifestyle, and relaxation techniques; (b) cognitive restructuring; (c) time management and goal setting; (d) assertiveness skills; and (e) evaluation and relapse prevention	Regular GP and OP consultations	Physical function; Depression; Anxiety; Stress; Sickness absence	7
Vente et al. [33] (grouped CBT)	Psychological	Netherlands	NB	41.2	16|12	17|9	16 weeks	12 × 2-h group CBT-based stress management therapies; Treatment protocols for both the individual and group SMT comprised five modules (a) psychoeducation, self-assessment of stressors and complaints, lifestyle, and relaxation techniques; (b) cognitive restructuring; (c) time management and goal setting; (d) assertiveness skills; and (e) evaluation and relapse prevent	Regular GP and OP consultations	Physical function; Depression; Anxiety; Stress; Sickness absence	7
Volker et al. [51]	Psychological	Netherlands	NB	44.2	54|77	36|53	52 weeks	Collaborative Occupational health care: 16 sessions of 5 online module: (1) psychoeducation, (2) a module aimed at cognitions regarding RTW while having symptoms (based on cognitive behavioural therapy [CBT] principles), (3) a module aimed at increasing problem-solving skills with problem-solving treatment (PST) exercises, (4) a module for pain and fatigue management and for reactivation, and (5) a module for relapse prevention; + individual discussion with OP	Usual care for CMD provided by OP	RTW, sick leave, depression, anxiety	5
Willert et al. [54]	Psychological	Denmark	NB	45	10|41	8|43	16 weeks	8 × 3-h group sessions with clinical psychologists with topics including (i) introduction to cognitive behaviour therapy, (ii) psychoeducation on stress, (iii) identifying dysfunctional thinking, (iv) modifying dysfunctional thinking, (v) communication and stress, (vi) communication skills training, (vii) implementing strategies at work, and (viii) review of techniques	No treatment for 3 months	Weeks of sick leave	5

*W-CBT* work-focused Cognitive Behavioral Therapy, *CBT-AU* Cognitive Behavioral Therapy as usual, *CSU* Care-as-usual, *CMD* Common Mental disorder

### Post- to Pre-treatment Effects of CBT-Based Intervention

#### Effects on Sick Leave in Reduced Days and People RTW in Number

**Fig. 2:**
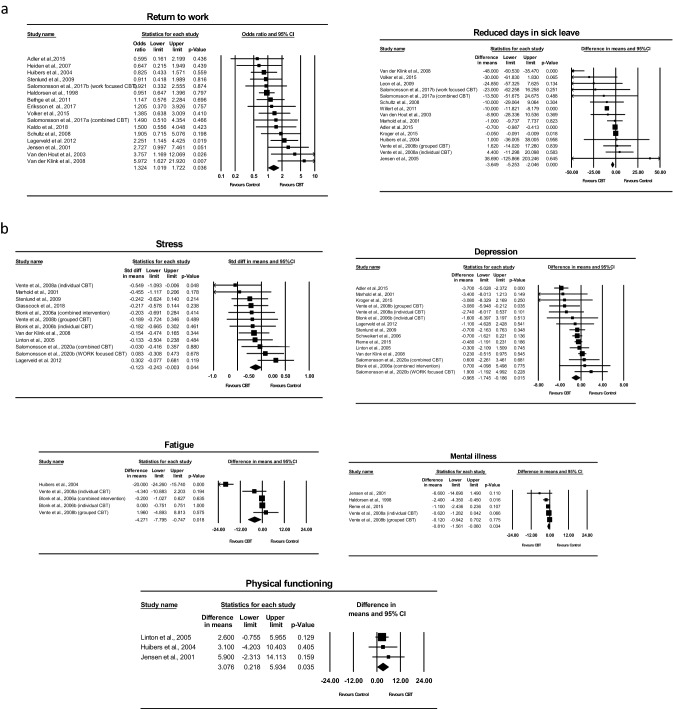
**a** Forest plots of the effects of CBT-based intervention on sick leave in reduced days and people RTW in number. **b** Forest plots of the effects of CBT-based intervention on mood symptoms (depression, anxiety and stress), fatigue, mental illness, physical function and working ability

Out of the 34 papers, 15 papers with 1727 participants investigated on reduction of sick leave, and the results showed that CBT-based intervention was more effective than the control condition in reducing sick leave in days with a mean reduction of − 3.649 (95% Cl − 5.253 to − 2.046; p < 0.001) and a moderate effect size of − 0.395 (95% Cl − 0.670 to − 0.120; p < 0.01). The heterogeneity of these studies was statistically significant (I^2^ = 92.991; p < 0.001) (Table2). Sixteen papers with 2298 participants reported on the number of people RTW. The results showed that CBT-based intervention was more effective than the control condition in facilitating people to RTW with a mean difference of 1.5 (95% Cl 1.019 to 1.722; p < 0.05). The heterogeneity of these studies was not statistically significant (I^2^ = 32.998, p > 0.05). The forest plots of the effect on sick leave in reduced days and numbers of people RTW are presented in Fig. 2a.

**Table 2:** Result of all variable analysis of included studies in meta-analysis

Continuous variables	Studies (n)	Participant (n)	Mean difference			Effect size			Publication bias
			MD (95% CI)	Q test	I^2^(%)	Effect size (95% CI)	Q test	I^2^(%)	Egger’s *t* value (95% CI)
Sick leave in reduced days	15	1727	− 3.649 (− 5.253, − 2.046) ***	199.729	92.991***	− 0.395(− 0.670, − 0.120)**	101.266	86.175***	1.796 (− 3.687, 0.355)
Working ability	8	1112	0.164 (− 0.221, 0.549)	192.564	96.365***	0.757 (− 0.788, 2.302)	736.096	99.049***	0.505 (− 33.797, 51.377)
Mental illness	5	1301	− 0.810 (− 1.561, − 0.060)*	7.208	44.510	− 0.196 (− 0.308, − 0.083)**	2.585	0.000	2.676(− 4.287, 0.371)
Physical function	3	364	3.076 (0.218, 5.934)*	0.532	0.000	0.223 (0.016, 0.430)*	0.498	0.000	1.363 (− 7.153, 8.872)
Stress	12	1233	− 0.722 (− 1.484, 0.041)	11.174	1.557	− 0.123 (− 0.243, − 0.003)*	10.335	0.000	1.643 (− 5.286, 0.799)
Depression	15	2870	− 0.965(− 1.745, − 0.186)*	36.354	61.490**	− 0.176 (− 0.322, − 0.031)*	32.654	57.126**	1.063 (− 2.258, 0.769)
Anxiety	12	2078	− 0.380 (− 0.816, 0.057)	8.885	0.000	− 0.089 (− 0.178, 0.001)	8.004	0.000	0.006 (− 1.481, 1.474)
Fatigue	5	390	− 4.271 (− 7.795, − 0.747)*	84.457	95.264***	− 0.376 (− 1.058, 0.306)	41.644	90.395***	1.220 (− 12.977, 5.786)

Categorical variables			Odds ratio			Publication bias
			Odds ratio (95% CI)	Q test	I^2^(%)	Egger’s *t* value (95% CI)
People RTW in number	16	2298	1.5 (1.019, 1.722)*	22.388	32.998	1.677 (− 0.381, 3.103)

*p < 0.05, **p < 0.01, ***p < 0.001

The subgroup analyses were performed to assess the effect of CBT on sick leave in reduced days. Table 3 suggested that CBT based intervention in the studies with the following characteristics had a significant effect on sick leave in reduced day: treatment delivered face-to-face (MD − 8.673, 95% CI − 15.550, − 1.797, p < 0.05), participants with higher education levels (> 12 years) (effect size − 0.923, 95% CI − 1.206, − 0.639, p < 0.001), utilization of rehabilitation services (MD − 10.095, 95% CI − 11.902, − 8.288, p < 0.001), stress management (MD − 3.498, 95% CI − 5.110, − 1.886, p < 0.001), and long treatment course (≥ 16 weeks) (MD 2.747, 95% CI − 4.169, − 1.326, p < 0.001). CBT-based intervention showed a better effect on sick leave in studies that included homework assignment (MD − 2.615, 95% CI − 4.017, − 1.213, p < 0.001), mood management (effect size − 0.926, 95% CI − 1.209, − 0.643), p < 0.001), long duration of sessions (≥ 90 min) (MD − 9.951, 95% CI − 17.633, − 2.269, p < 0.05), combined CBT (MD − 14.785, 95% CI − 22.898, − 6.672, p < 0.001) and those delivered in group sessions (effect size − 9.476, 95% CI − 11.247, − 7.704, p < 0.001).

**Table 3:** Sick leave in reduced days subgroup analysis

Subgroups	Studies (n)	Participants (n)	Sick leave in reduced days: post- to pre- treatment effect				Q test	I^2^(%)
			Mean difference (95% CI)	P between	Effect size (95% CI)	P between		
Delivery method of CBT				0.912		0.000		
Face to face	11	1057	− 8.673 (− 15.550, − 1.797)*		− 0.056 (− 0.097, − 0.014)**		174.075	94.255***
Remote	3	568	− 13.489 (− 34.837, 7.859)		− 0.704 (− 0.991, − 0.417)***		5.379	62.815
Education level				0.541		0.000		
< 9 years	3	353	− 8.894 (− 31.002, 13.214)		− 2.923 (− 11.337, 5.492)		3.212	37.725
9–12 years	6	722	− 16.159 (− 38.885, 6.567)		− 0.051 (− 0.092, − 0.009)		59.093	91.539***
> 12 years	4	387	− 2.949 (− 10.506, 4.608)		− 0.923 (− 1.206, − 0.639)***		98.315	96.949***
Reasons for sick leave				0.000		0.000		
MSK	5	398	− 4.430 (− 11.594, 2.734)		− 4.430 (− 11.594, 2.734)		2.906	0.000
Psychological	7	754	− 0.809 (− 2.029, 0.412)		− 0.064 (− 0.105, − 0.023)**		77.681	92.276***
Others	3	473	− 10.039 (− 11.854, − 8.223)***		− 10.039 (− 11.854, − 8.223)***		1.854	0.000
Rehabilitation services utilization				0.000		0.000		
No	10	1043	− 0.834 (− 2.024, 0.356)		− 0.064 (− 0.105, − 0.023)**		78.772	88.575***
Yes	5	684	− 10.095 (− 11.902, − 8.288)***		− 10.095 (− 11.902, − 8.288)***		2.658	0.000
Support from supervisor				0.479		0.000		
No	11	1352	− 5.193 (− 11.143, 0.758)		− 0.931 (− 1.214, − 0.648)***		106.116	90.576***
Yes	4	375	− 16.920 (− 48.858, 15.019)		− 0.051 (− 0.092, − 0.009)*		57.263	94.761***
Mood management				0.243		0.000		
No	6	697	− 17.856 (− 40.277, 4.566)		− 0.051 (− 0.092, − 0.009)*		61.711	
Yes	9	1030	− 3.984 (− 10.232, 2.264)		− 0.926 (− 1.209, − 0.643)***		102.042	91.898***
Stress management				0.168		0.054		92.160***
No	4	503	− 12.340 (− 24.818, 0.138)		− 12.340 (− 24.818, 0.138)		1.730	
Yes	11	1224	− 3.498 (− 5.110, − 1.886)***		− 0.069 (− 0.110, − 0.028)**		194.284	0.000
Homework assignment				0.070		0.000		94.853***
No	5	629	− 21.866 (− 42.679, − 1.054)*		− 29.076 (− 37.812, − 20.340)***		17.480	
Yes	10	1098	− 2.615 (− 4.017, − 1.213)***		− 0.068 (− 0.109, − 0.027)**		139.896	77.116**
Psychological education				0.024		0.000		93.567***
No	5	676	− 21.050 (− 39.009, − 3.090)*		− 10.824 (− 12.607, − 9.040)***		36.040	
Yes	10	1051	− 0.364 (− 0.939, 0.211)		− 0.063 (− 0.104, − 0.022)**		23.956	88.901***
Relapse prevention				0.282		0.000		62.432**
No	11	1232	− 5.511 (− 11.893, 0.872)		− 0.930 (− 1.214, − 0.647)***		104.900	
Yes	4	495	− 17.033 (− 37.015, 2.948)		− 0.051 (− 0.092, − 0.009)*		58.540	90.467***
Interpersonal strategies				0.001		0.000		94.875***
No	7	940	− 14.705 (− 22.971, − 6.438)***		− 0.955 (− 1.238, − 0.672)***		156.893	
Yes	8	787	− 0.050 (− 0.091, − 0.009)*		− 0.050 (− 0.091, − 0.009)*		4.431	96.176***
Intervention type				0.000		0.000		0.000
Single CBT	8	704	− 0.050 (− 0.091, − 0.009)*		− 0.050 (− 0.091, − 0.009)*		4.167	
Combined CBT	7	1023	− 14.785 (− 22.898, − 6.672)***		− 0.956 (− 1.239, − 0.673)***		157.085	0.000
Duration of session				0.007		0.000		96.180***
< 90 min	5	655	− 0.702 (− 0.989, − 0.415)***		− 0.702 (− 0.989, − 0.415)***		3.452	
≥ 90 min	7	577	− 9.951 (− 17.633, − 2.269)*		− 0.056 (− 0.097, − 0.014)**		171.974	0.000
Treatment length in weeks				0.353		0.000		96.511***
< 16 weeks	4	385	− 17.493 (− 48.574, 13.587)		− 15.391 (− 22.110, − 8.672)***		37.282	
≥ 16 weeks	11	1342	− 2.747 (− 4.169, − 1.326)***		− 0.068 (− 0.109, − 0.027)**		142.468	91.953***
Combined with mood symptoms				0.018		0.000		92.981***
No	10	1162	− 11.945 (− 21.505, − 2.385)*		− 10.122 (− 11.844, − 8.400)***		47.412	
Yes	5	565	− 0.367 (− 0.986, 0.253)		− 0.063 (− 0.104, − 0.022)**		21.316	81.017***
Treatment form				0.380		0.000		81.235***
Individual session	10	1344	− 0.962 (− 2.211, 0.286)		− 0.064 (− 0.105, − 0.023)**		84.319	
Group session	4	299	− 5.308 (− 12.622, 2.006)		− 9.476 (− 11.247, − 7.704)***		6.196	89.326
Mixed	1	84	− 8.900 (− 28.336, 10.536)		− 8.900 (− 28.336, 10.536)		0.000	51.582
Study design				0.002		0.000		0.000
Non− RCT	1	26	− 0.050 (− 0.091, − 0.009)*		− 0.050 (− 0.091, − 0.009)*		0.000	
RCT	14	1701	− 9.984 (− 16.205, − 3.763)**		− 0.956 (− 1.239, − 0.673)***		161.161	0.000

*p < 0.05, **p < 0.01, ***p < 0.001

#### Effect on Psychological Condition (Mental Illness, Stress, Anxiety, and Depression)

12 studies with a total sample of 1233 and 2078 participants were included in the random-effects meta-analysis for stress and anxiety respectively, while 5 studies with a total sample size of 1301 were analysed for mental illness. The result in Table 2 showed no significant difference between CBT-based intervention and control conditions for anxiety (MD − 0.380, 95% CI − 0.816, 0.057, p > 0.05). CBT-based interventions were more effective in managing stress in contrast to the control condition with a small effect size of − 0.123 (95% Cl − 0.243, − 0.003, p < 0.05). CBT-based interventions were also more effective in managing mental illness compared to the control condition with mean reduction of − 0.810 (95% Cl − 1.561, − 0.060; p < 0.05), with a small effect size of − 0.196 (95% Cl − 0.308, − 0.083, p < 0.01).

The effect on depression was analysed in 15 studies that had a total sample of 2870 participants. CBT-based interventions were more effective for depression management compared to the control condition with mean reduction of depression of -0.965 (95% Cl − 1.745, − 0.186, p < 0.05), and a small effect size of − 0.176 (95% Cl − 0.322, − 0.031, p < 0.05) (Table 2). The heterogeneity for these studies was statistically significant (I^2^ = 61.490; p < 0.01) (Table 2). Subgroup analyses also showed that CBT-based interventions with the following characteristics had a significant effect on depression symptoms (Table 4): participants with higher education levels (> 12 years) (MD − 0.349, 95% CI − 0.667, − 0.031, p < 0.05), rehabilitation services (MD − 0.132, 95% CI − 0.252, − 0.012, p < 0.05), stress management (MD − 0.222, 95% CI − 0.424, − 0.021, p < 0.05), homework assignments (MD − 0.382, 95% CI − 0.626, − 0.138, p < 0.01), psychological education (MD − 0.209, 95% CI − 0.384, − 0.034, p < 0.05), interpersonal skills (MD − 0.110, 95% CI − 0.213, − 0.007, p < 0.05), long treatment course (≥ 16 weeks) (MD − 0.210, 95% CI − 0.409, − 0.012, p < 0.05) and long duration of sessions (≥ 90 min) (MD − 0.153, CI 95% − 0.298, − 0.008, p < 0.05), treatment delivered in group sessions (MD − 0.197, 95% CI − 0.383, − 0.010, p < 0.05) in forms of combined CBT (effect size − 0.164, 95% CI − 0.267, − 0.061, p < 0.01). CBT-based interventions showed a better effect on depression when treatment was delivered remotely (MD − 0.892, 95% CI − 1.228, 0.556, p < 0.001). The forest plots of the effect on the psychological condition are presented in Fig. 2b**.**

**Table 4:** Depression subgroup analysis

Subgroups	Studies (n)	Participants (n)	Depression: post- to pre- treatment effect				Q test	I^2^(%)
			Mean difference (95% CI)	P between	Effect size (95% CI)	P between		
Delivery method of CBT				0.000		0.000		
Face to face	14	2703	− 0.103 (− 0.191, − 0.014)*		− 0.103 (− 0.191, − 0.014)*		12.811	0.000
Remote	1	167	− 0.892 (− 1.228, − 0.556)***		− 0.892 (− 1.228, − 0.556)***		0.000	0.000
Education level				0.143		0.066		
< 9 years	1	36	− 0.481 (− 1.144, 0.181)		− 0.481 (− 1.144, 0.181)		0.000	0.000
9–12 years	6	702	0.004 (− 0.154, 0.161)		0.004 (− 0.154, 0.161)		4.512	0.000
> 12 years	6	1607	− 0.349 (− 0.667, − 0.031)*		− 0.252 (− 0.378, − 0.127)***		20.744	75.896**
Reasons for sick leave				0.988		0.998		
MSK	2	152	− 0.178 (− 0.547, 0.191)		− 0.162 (− 0.485, 0.162)		1.175	14.899
Psychological	12	2309	− 0.179 (− 0.364, 0.006)		− 0.153 (− 0.251, − 0.054)**		31.474	65.051**
Rehabilitation services utilization				0.600		0.608		
No	12	1134	− 0.198 (− 0.414, 0.018)		− 0.177 (− 0.299, − 0.055)**		32.177	65.814**
Yes	3	1736	− 0.132 (− 0.252, − 0.012)*		− 0.132 (− 0.252, − 0.012)*		0.213	0.000
Support from supervisor				0.601		0.229		
No	13	2676	− 0.193 (− 0.349, − 0.037)*		− 0.170 (− 0.259, − 0.081)***		29.568	59.416**
Yes	2	194	− 0.057 (− 0.542, 0.428)		0.019 (− 0.276, 0.315)		1.639	38.986
Use of complimentary therapy				0.880		0.911		
No	13	2325	− 0.183 (− 0.362, − 0.004)*		− 0.151 (− 0.248, − 0.055)**		32.629	63.223**
Yes	2	545	− 0.163 (− 0.346, 0.019)		− 0.163 (− 0.346, 0.019)		0.012	0.000
Stress management				0.236		0.129		
No	4	729	− 0.077 (− 0.208, 0.055)		− 0.077 (− 0.208, 0.055)		2.198	0.000
Yes	11	2141	− 0.222 (− 0.424, − 0.021)*		− 0.211 (− 0.324, − 0.098)***		28.154	64.481
Homework assignment				0.016		0.001		
No	7	2103	− 0.055 (− 0.159, 0.048)		− 0.055 (− 0.159, 0.048)		5.341	0.000
Yes	8	767	− 0.382 (− 0.626, − 0.138)**		− 0.371 (− 0.524, − 0.218)***		16.078	56.462*
Psychological education				0.329		0.348		
No	2	577	− 0.059 (− 0.303, 0.185)		− 0.081 (− 0.255, 0.093)		1.738	42.463
Yes	13	2293	− 0.209 (− 0.384, − 0.034)*		− 0.177 (− 0.275, − 0.079)***		30.035	60.047**
Relapse prevention				0.965		0.488		
No	12	2640	− 0.181 (− 0.342, − 0.020)*		− 0.164 (− 0.254, − 0.074)***		28.703	61.677**
Yes	3	230	− 0.171 (− 0.593, 0.252)		− 0.064 (− 0.333, 0.206)		3.470	42.361
Interpersonal strategies				0.452		0.131		
No	3	744	− 0.310 (− 0.822, 0.201)		− 0.253 (− 0.408, − 0.099) **		19.382	89.681***
Yes	12	2126	− 0.110 (− 0.213, − 0.007)*		− 0.110 (− 0.213, − 0.007)*		10.995	0.000
Intervention type				0.762		0.727		
Single CBT	9	696	− 0.145 (− 0.320, 0.030)		− 0.131 (− 0.285, 0.023)		9.846	18.752
Combined CBT	6	2174	− 0.190 (− 0.429, 0.048)		− 0.164 (− 0.267, − 0.061)**		22.686	77.960***
Duration of session				0.283		0.005		
< 90 min	4	352	− 0.377 (− 0.839, 0.084)		− 0.472 (− 0.692, − 0.253)***		12.397	75.801
≥ 90 min	7	955	− 0.153 (− 0.298, − 0.008)*		− 0.150 (− 0.285, − 0.014)*		6.437	6.785
Treatment length in weeks				0.387		0.400		
< 16 weeks	4	729	− 0.098 (− 0.256, 0.059)		− 0.099 (− 0.253, 0.054)		3.096	3.098
≥ 16 weeks	11	2141	− 0.210 (− 0.409, − 0.012)*		− 0.179 (− 0.282, − 0.076)**		28.850	65.338**
Combined with mood symptoms				0.787		0.831		
No	9	1114	− 0.144 (− 0.268, − 0.020)*		− 0.144 (− 0.268, − 0.020)*		7.803	0.000
Yes	6	1756	− 0.190 (− 0.499, 0.119)		− 0.163 (− 0.281, − 0.045)**		24.805	79.842***
Treatment form				0.951		0.869		
Individual session	9	1941	− 0.157 (− 0.395, 0.081)		− 0.138 (− 0.247, − 0.030)*		29.013	72.426***
Group session	5	520	− 0.197 (− 0.383, − 0.010)*		− 0.197 (− 0.383, − 0.010)*		3.359	0.000
Mixed	1	409	− 0.158 (− 0.365, 0.050)		− 0.158 (− 0.365, 0.050)		0.000	0.000
Study design				0.922		0.979		
Non-RCT	2	194	− 0.158 (− 0.465, 0.149)		− 0.158 (− 0.465, 0.149)		0.645	0.000
RCT	13	2676	− 0.175 (− 0.337, − 0.013)*		− 0.154 (− 0.243, − 0.065)**		32.008	62.509***

*p < 0.05, **p < 0.01, ***p < 0.001

#### Effect on Physical Condition (Working Ability, Fatigue, and Physical Function)

8 studies with 1112 participants were analysed for working ability. The result in Table 2 showed no significant difference between CBT-based intervention and control conditions for working ability (MD 0.164, 95% Cl − 0.221, 0.549, p > 0.05). 5 studies with 390 participants investigated fatigue while 3 studies with 364 participants investigated physical functioning. CBT-based interventions were more effective in managing fatigue and improving physical functioning when compared to the control condition with mean difference of − 4.271 (95% Cl − 7.795, − 0.747, p < 0.05) and 3.076 (95% Cl 0.218, 5.934, p < 0.05), respectively, with moderate effect sizes of − 0.376 (95% Cl − 1.058, 0.306, p < 0.05) for fatigue and 0.223 (95% Cl 0.016, 0.430, p < 0.05) for physical functioning. Forest plots of the effects of CBT-based intervention on the physical condition are presented in Fig. 2C.

### Publication bias

The Egger test in Table 5 suggested publication bias is minimal for all outcome variables (p > 0.05). The overall results for all outcome variables were not significantly altered when any one study is removed, indicating that the results were not overly distorted by any paper.

**Table 5:** Egger’s regression analysis on publication bias of included studies

Variables	T value	95% Cl	P-value
Sick leave in reduced days	1.796	− 3.687, 0.355	0.098
Working ability	0.505	− 33.797, 51.377	0.632
Mental illness	2.676	− 4.287, 0.371	0.075
Physical function	1.363	− 7.153, 8.872	0.403
Stress	1.643	− 5.286, 0.799	0.131
Depression	1.063	− 2.258, 0.769	0.307
Anxiety	0.006	− 1.481, 1.474	0.995
Fatigue	1.220	− 12.977, 5.786	0.309
People RTW in number	1.677	− 0.381, 3.103	0.116

## Discussion

This meta-analysis aimed to determine whether CBT-based interventions are effective to increase the number of people who return to work, reduce sick leave days, and improve mood symptoms, working ability, and physical function in employees with sick leave. Our results confirm that CBT-based intervention significantly increased the number of people who RTW and improved physical function. The intervention group also had a reduced amount of sick leave time (days) as well as reduced mental illness, depression, stress, and fatigue symptoms. While previous studies mainly focused on the ability of CBT to treat certain conditions, this is one of the first meta-analyses to comprehensively examine the effectiveness that CBT has on multiple outcomes.

Our study found that CBT-based interventions significantly increased the number of participants who RTW and reduced the number of days spent on sick leave. Previous studies have also showcased the ability of CBT to facilitate RTW [20]. The improvements in sick leave duration and willingness to RTW seen in this study may be due to the enhanced mental health of participants. Our results indicate that individuals who have undergone CBT experienced decreased mental illness, depression, stress, and fatigue symptoms, which is consistent with another study conducted by Salomonsson et al. investigating the effects of CBT for patients on sick leave with common mental health disorders [63]. Several reasons exist as to why improving mental health with CBT leads to greater sick leave reduction. Firstly, the medical treatment of MSK pain and mental health disorders alone may be insufficient in reducing sick leave. To address this, CBT provides psychological-based treatment and work attitude adjustment in conjunction with the management of the physical causes of work absence [16]. CBT improves patient cognition and thought processes that underly their reasons for taking sick leave, allowing patients to cope with their circumstances [17]. This is supported by our study’s subgroup analysis findings that CBT was more effective in reducing sick leave when stress management was incorporated in the CBT process. By instilling positive attitudes towards work and reducing stress and burnout, individuals may be more eager to RTW after CBT, thus reducing sick leave duration. Equally, other studies have shown that CBT is effective in reducing stress and insomnia which can enhance energy levels and willingness to RTW [18]. CBT leads to a reduction in psychiatric symptoms and improvements in patient satisfaction [63]. Overall, the improvement of mental health due to CBT may lead to reduced sick leave duration since individuals are more likely to RTW faster if they are less stressed and fatigued.

Apart from stress management, our subgroup analyses further identified several factors that contributed to the effectiveness of CBT on reduced sick leave in days. Combined CBT that is work focused intervention in conjunction with other rehabilitations significantly reduced sick leave duration in comparison to CBT-only interventions, allowing employees to RTW faster. We speculate that despite CBT managing employees’ psychosocial complaints, it might not be directly correlated to work resumption as their working ability and subjective health were unaddressed. Meanwhile, combined CBT that utilised various intervention components not only focused on work problem solving bur also facilitated solving other health problems. The important components of combined CBT evidenced in our subgroup analysis were homework assignments and rehabilitation services. The importance of homework assignments could be explained by their effectiveness in managing symptoms between sessions and adapting to new skills. We speculate that since CBT was generally not a therapy delivered daily, homework assignments allowed individuals to practice their coping strategies between sessions and maintain their skills when they resumed to workplace. Our finding was consistent with a study conducted by Van der Klink et al. who investigated the effectiveness of CBT combined with stress management and homework assignment on sick leave in employees with mental health disorders [50]. Rehabilitation services could provide work-based education and physical training to allow employees to be mentally and physically prepared for RTW [35]. An example of rehabilitation would be low-intensity exercises for MSK-related injuries that allow employees to gradually regain their functional abilities with a reduced chance of further injury. Another example of rehabilitation services is the use of psychosocial strategies including relapsing preventions to support participants at the workplace [60]. The finding suggested that the work-focused components such as homework assignments and rehabilitation services focused on solving health problems that are frequently encountered at work, consequently allowing employees to restore self-esteem. Conversely, our subgroup analysis has also shown that there was no significant difference in reduced sick leave in days with the use of workplace-focused components including supervisor support and relapse prevention strategies. Firstly, we speculate that although supervisor support may provide appropriate workplace adjustments, employees may be reluctant to express their needs at the workplace due to self-stigma. This is consistent with a study conducted by Dalgaard et al. which demonstrated that only 10% of the participants wanted their supervisors and psychologists to participate in their direct workplace interventions [52]. Secondly, relapse prevention may reduce the frequency and duration of relapse episodes by developing positive attitudes and strategies when facing triggers in the workplace. However, its effectiveness may not be achieved if performed outside the workplace since there is a lack of context during the intervention. We speculate that the effectiveness of relapse prevention may be limited if it was delivered while employees were still on sick leave. Overall, it can be concluded that the effectiveness of CBT in facilitating RTW may be multifactorial and requires both workplace and health interventions. More research investigating the work-focused components of combined CBTs including relapse prevention and supervisor support would be required to evaluate their effectiveness.

### An Update of the Recent Studies

A recent RCT conducted by Hoff et al. compared an integrated intervention (INT) with a non-integrated group and a care-as-usual (CAU) group [64]. The non-integrated method was referred to as Mental Healthcare (MHC) which was based on the conventional CBT while the INT integrated ‘healthcare and vocational rehabilitation’. The study suggested no difference in effect on the time to RTW between the MHC and the CAU group (98.3% Cl 0.88, 1.55, p = 0.196). This result is opposed to our findings that CBT-based interventions significantly reduced the number of days spent on sick leave. The insignificant difference in time to RTW in this RCT could be explained by many factors. Firstly, the staff only received a short period of training for standard CBT that may comprise the quality of treatment delivered to patients. Secondly, the insignificance may be related to the poor standard of the sessions since many studies have evidenced poor healthcare in primary care [65, 66]. Lastly, this RCT utilised standard CBT instead of work-focused CBT which has been found to be more effectively in facilitating RTW based on our findings. This RCT also found that the time to RTW in the MHC group was 0.79 times that in the INT group (98.3% Cl 0.6, 1.05, p = 0.044), suggesting an integrated intervention would be more effectively than a single intervention. Similarly, our review also suggested that combined CBT which is work focused intervention in conjunction with other rehabilitations significantly reduced sick leave duration in comparison to CBT-only interventions, allowing employees to RTW faster.

Additionally, certain forms of CBT-based intervention utilised in this study allowed sick leave days to be further reduced. These include ≥ 90 min session duration (where participants had 9.95 days of reduced sick leave on average compared to 0.7 days for sessions < 90 min) and ≥ 16 weeks treatment length, indicating that increasing intervention exposure time improves outcomes. However, this will require further research as there is currently a lack of studies that investigate the relationship between CBT duration and sick leave reduction. We also found that group-delivered CBT was more effective than individualised CBT in reducing sick days and depression. This could be explained by group CBT emphasising the influence of social factors on individual behaviour. Participants can access more opportunities for positive peer modelling and the normalisation of their symptoms through communication with group members [67]. CBT also reduced sick leave duration more when performed face-to-face compared to remote delivery (including via phone, internet, and online modules). We speculate that this may be due to the increased engagement and accountability of patients in face-to-face settings. Over the last two decades, hundreds of RCTs have showcased that internet-based CBT produces promising outcomes. However, the control groups were usually care-as-usual, preventing comparisons between different CBT delivery methods [68]. To address this, a 2018 meta-analysis compared the effectiveness of internet-based and face-to-face CBT for psychiatric and somatic disorders in 1418 participants from 20 included articles [68]. Overall effects were found to be equivalent between the two delivery methods [68]. Equally, a 2020 RCT found that internet-based CBT was non-inferior and cheaper than face-to-face CBT for anxiety improvement [69]. More research with larger sample sizes is required to establish further comparisons.

Several other key issues exist regarding the effectiveness of CBT. Our study found that working ability was not significantly improved by CBT despite reduced sick leave days, which increases the concern of presenteeism. Although individuals returned to work earlier, they may have had ongoing issues that prevented them from working at their full potential. Another problem is that sick leave was only reduced by 3.65 days on average, which needs to be considered in the context of the time and cost of implementing CBT [70].

Overall, while the findings of our study support the benefits of utilising CBT to improve mental health and enable earlier RTW, more research is required to determine the optimal timing and cost-effectiveness of CBT implementation.

### Limitations, Strengths, and Implications

Despite the variety of papers considered in this study, there are several limitations. Firstly, there was high heterogeneity among the RCTs used which can be due to the fact that many of the outcomes being studied were measured on different scales by the authors of the RCTs that comprise the dataset for this study. Secondly, the outcome variables measured at different ending points may alter the validity of the result. Although we aimed to include the outcomes at the end point of the intervention, some outcomes were only measured a few months after the post-intervention which may alter the effectiveness of the intervention. In addition, although most RCTs included in this systematic review and meta-analysis were based on a CBT manual, the environmental settings and the focus of different CBT components varied. Therefore, the overall effectiveness of CBT on RTW would depend on the skills of practitioners, the dedication of patients to change, and whether it is also work-focused rather than only health-focused.

This study provides promising results for the utilisation of CBT in those unable to work due to MSK and psychiatric illnesses. By reducing sick leave, individuals can RTW sooner and increase the productivity of their workplaces [71]. The finding of decreased mental illness, depression, stress, and fatigue after CBT treatment has significant implications for the health of workers and the prevention of burnout [72]. More research is needed to assess the effectiveness of increasing CBT duration, comparing face-to-face and remote delivery methods, and whether CBT is better as a preventative measure used in the early stages of illness before sick leave commencement [73].

## Conclusion

Our findings demonstrate that there was a significant reduction in the length of sick leave and improving the rate of RTW of employees. The subgroup analysis suggested that CBT-based intervention in the studies with the following characteristics had a better effect on sick leave in reduced days: treatment delivered face-to-face, utilization of rehabilitation services, stress management, homework assignment and long treatment course (≥ 16 weeks). We did not find any significant difference between the CBT-based interventions and the control in reducing anxiety, and working ability but CBT-based interventions were more effective in managing stress, fatigue, mental illness, physical function, and depression compared to the control condition. Subgroup analysis results demonstrate that CBT-based interventions with the following characteristics had a better effect on depression symptoms: participants with higher education levels (> 12 years, longer treatment courses (≥ 16 weeks), and treatment delivered in group sessions. CBT-based interventions showed a better effect on depression when they used the following components: rehabilitation services, homework assignments, and psychological education. Overall, CBT-based interventions are a good option for promoting RTW and reducing the length of sick leaves in employees.

## Data Availability

The datasets generated during and/or analyzed during the current study are available from the corresponding author on reasonable request.
